# CpG Methylation in Aging: Trajectories of Individual Sites

**DOI:** 10.1093/geroni/igaa057.430

**Published:** 2020-12-16

**Authors:** Margarita Meer, Morgan Levine

**Affiliations:** 1 Yale School of Medicine, New Haven, Connecticut, United States; 2 Yale University School of Medicine, New Haven, Connecticut, United States

## Abstract

Age-related changes in methylation in a set of genomic CpGs have been shown to form a kind of molecular clocks of aging – DNA methylation (DNAm) clocks. These markers are usually based on a small set of CpGs in every case, but 1) they rarely overlap between different clocks and 2) they are interchangeable, meaning that one can remove all clock sites from a data set and make a new clock of similar precision selecting a new set from the remaining sites. Nonetheless, only a fraction of CpG sites would be suitable for DNAm clocks. We performed an extensive analysis of all CpG sites aging behavior. Previous studies were focused on identifying positions where changes in DNAm correlate with age, but in this case, some of CpGs where DNAm changes occur in a non-linear way can be overlooked. We assessed the aging trajectory of every CpG, clustered CpGs by the type of aging behavior and applied a machine learning approach to construct a new kind of DNAm clocks based on the DNAm of these clusters. Since every cluster is composed of multiple CpGs, it makes this marker resistant to a common problem of missing data. Using blood, brain, skin, colon and liver samples we were also able to investigate tissue specificity of CpGs trajectories.

